# Quality of Life in Adults with Congenital Heart Disease: Insights from a Tertiary Centre

**DOI:** 10.3390/jcm14207451

**Published:** 2025-10-21

**Authors:** Polona Kacar, Melita Flander, Katja Prokselj

**Affiliations:** 1 Department of Cardiology, University Medical Centre Ljubljana, 1000 Ljubljana, Slovenia; 2 Faculty of Medicine, University of Ljubljana, 1000 Ljubljana, Slovenia; 3 Family Medicine Clinic, Idrija Medical Centre, 5280 Idrija, Slovenia

**Keywords:** adult congenital heart disease, quality of life

## Abstract

**Objective:** As the survival of individuals born with congenital heart disease (CHD) improves into adulthood, the focus has shifted from traditional clinical outcomes to patient-reported outcome measures that better reflect the impact of the disease on daily life. Our aim was to assess the quality of life (QoL) of adult patients with congenital heart disease (ACHD) followed in a tertiary centre and to evaluate the parameters that influence QoL in this population. **Methods:** This cross-sectional observational study included patients followed up at the national referral ACHD centre between April and September 2022. Sociodemographic and clinical data were collected from medical records and self-report questionnaires. Quality of life (QoL) was assessed using the validated Short Form–36 (SF-36) and Euro Quality of Life–5 Dimension (EQ-5D) questionnaires, including the EQ Visual Analogue Scale (VAS). **Results:** A total of 123 ACHD patients were included (median age 34 (29–41) years; 43.9% male). Most participants had moderate CHD (61%), and 14.6% were cyanotic. Overall, SF-36 Physical Component Summary scores were higher than Mental Component Summary scores. Almost half of the patients (48.8%) reported no problems in all five domains of the EQ-5D, with most problems reported in anxiety/depression domain. Patients with severe CHD, cyanosis, or HF reported lower QoL scores across multiple SF-36 domains, particularly general health, role–physical, and physical functioning domains. **Conclusions:** QoL among ACHD patients in our cohort was generally high in most domains as assessed by the SF-36 and EQ-5D. Patients with HF reported lower QoL scores, emphasizing the importance of close clinical follow-up and the need for tailored QoL assessment tools for this complex population.

## 1. Introduction

Congenital heart disease (CHD) is the most common congenital disease with an estimated prevalence of 1% [1]. Due to recent advances in surgical techniques and improved medical treatment, more than 90% of all individuals born with CHD now survive into adulthood [2]. Nevertheless, these patients require lifelong medical care as many of them face residual problems.

Healthcare outcomes are most commonly measured by morbidity, mortality, and functional status. However, healthcare professionals are becoming increasingly aware of the importance of patient-reported outcome measures to capture not only physical but also psychological dimensions of disease, as well as mental health, physical well-being, illness perception, anxiety, and general health status [3]. Most patients with CHD transition to adult congenital heart disease (ACHD) clinics in early adulthood when they are still relatively young and free of most cardiovascular and age-related comorbidities. Consequently, they may appear to be healthier than their older peers, based on functional status or other clinical outcome measures. However, they often face challenges such as reproductive and sexual health concerns, employment issues, and uncertainty about prognosis, which can significantly affect their daily lives [4,5,6]. Healthcare professionals should be aware of these issues and monitor them regularly using appropriate health-related quality of life (QoL) questionnaires, as these tools can reveal the patient’s true well-being and the real impact of medical or surgical treatment—information that may not be apparent from objective health parameters and disease status alone.

For this reason, QoL in ACHD is increasingly being studied [7]. Although the literature indicates that patients with CHD generally have a lower QoL than healthy individuals—particularly when QoL is assessed in terms of physical functioning—some studies suggest that they report higher life satisfaction compared with the healthy population [8,9,10,11]. The latter may be partially explained by a stronger sense of coherence observed in ACHD patients [12,13].

However, when examining factors that influence QoL in this population, existing evidence remains inconsistent. Some studies report no association between QoL and the severity of CHD or residual lesions, while others suggest that patients with more complex conditions, including cyanotic heart disease, experience poorer QoL [14,15,16]. These conflicting findings highlight the need for further research into the determinants of QoL in ACHD patients.

We aimed to assess the QoL of ACHD patients followed up in a tertiary centre and to evaluate the parameters influencing QoL in this population.

## 2. Methods

This is a cross-sectional observational study. Adult patients followed up at the University Medical Centre Ljubljana, a national reference centre for ACHD, were invited to participate during their regular outpatient visit from April to September 2022. Inclusion criteria were patients aged 18 years or older with CHD; exclusion criteria were syndromic condition (e.g., Down’s syndrome or Marfan syndrome), lower cognitive ability, patients diagnosed with CHD in adulthood (age ≥ 18 years), and a follow-up time in an outpatient clinic of less than 1 year. Lower cognitive ability was defined based on documented intellectual disability or cognitive impairment in medical records, or an inability to independently complete the questionnaire despite assistance, in order to ensure the validity of self-reported data. Cyanosis was defined as an arterial oxygen saturation of <90% at rest, measured once during the study visit. Heart failure was defined as a left or right ventricular ejection fraction below 50%, or by a prior prescription of therapy for heart failure.

Sociodemographic data were obtained from available medical records and by a self-report questionnaire, including marital status, number of children, education level, and employment status. Clinical data were obtained from available medical records. CHD was classified as mild, moderate, or severe according to the ESC ACHD guidelines [17].

The study was conducted according to the guidelines of the Declaration of Helsinki and approved by Slovenian National Ethics Committee (Protocol No.: 0120-63/2022/3; date of approval: 19 April 2022). Informed consent was obtained from each participant.

### 2.1. Measures

QoL was assessed using the Short Form–36 (SF-36) and Euro Quality of Life–5 Dimension (EQ-5D) questionnaires. SF-36 is a validated patient-reported outcome measure consisting of 36 items that evaluate eight domains: physical functioning, role limitations due to physical health, bodily pain, general health perception, vitality, social functioning, role limitations due to emotional problems, and mental health. The scores for each domain range from 0 to 100, with higher scores indicating better perceived health. The domains can also be summarized into two composite scores: the Physical Component Summary (PCS) and the Mental Component Summary (MCS) [18].

EQ-5D is a standardized instrument consisting of five domains: mobility, self-care, usual activities, pain/discomfort, and anxiety/depression. Each domain is rated on five levels of severity (1–5) for each domain, with 1 indicating no problems and 5 indicating severe problems. The results were reported as raw scores to directly illustrate the distribution of patient-reported difficulties across the different health dimensions. These dimensions yield 3125 (5^5^) theoretically possible health states, which can be converted into a single weighted index score. In the Slovenian value set, index scores range from −1.09 to 1.00 [19]. In addition, patients rated their overall health status on the EQ Visual Analogue Scale (VAS), ranging from 0 (worst imaginable health status) to 100 (best imaginable health status) [20,21].

### 2.2. Statistical Analysis

The normal distribution of variables was tested with the Kolmogorov–Smirnov test. Normally distributed continuous variables are presented as mean value ± standard deviation and non-normally distributed continuous variables as median (Q1–Q3). Categorical variables are presented as numbers and percentages. The two-sample *t*-test and Mann–Whitney U-test were used to compare normally and non-normally distributed continuous variables, respectively. Comparisons between several groups of continuous variables were performed with the ANOVA test for normal and the Kruskal–Wallis test for non-normal distribution. For both tests, we used Bonferroni post hoc analysis. Categorical variables were analysed using the χ^2^ test. Correlation between continuous variables was performed using Pearson correlation for normal distribution and Spearman correlation for non-normal distribution. A *p* value < 0.05 was considered statistically significant. Statistical analysis was performed using IBM SPSS Statistics, version 26.

## 3. Results

### 3.1. Baseline Characteristics

We included 123 consecutive ACHD patients with median age of 34 years (29–41 years); 54 (43.9%) were male (Table 1). There were no non-responders, and all participants completed the questionnaires fully. More than two-thirds of the patients were either married or in a long-term relationship (66.7%), and more than half of the patients had at least one child (52%). Most patients had an education level greater than high school (52%), and almost all were employed (80.5%).

More than half of the patients had moderate CHD (61%), and 18 (14.6%) were cyanotic (Table 2). Most patients had normal left ventricular ejection fraction (89.3%) and normal right ventricular systolic function (73.8%). Only three patients had severely reduced right ventricular systolic function (2.5%).

### 3.2. Quality of Life

The results of the SF-36 questionnaire are presented in Table 3. The Physical Component Summary (PCS) scores were higher than the Mental Component Summary (MCS) scores, with the lowest scores reported in the domains of vitality and general health.

Almost half of the patients (48.8%) reported having no problems in all five domains of the EQ-5D questionnaire, and only four (3.3%) patients reported moderate problems or more in at least two domains (Table 3). Problems were most frequently reported in the anxiety/depression domain and least commonly in the self-care domain. The median EQ-5D index score was 1.000 (0.943–1.000). The overall median for EQ-VAS was 80 (70–90).

### 3.3. Correlations Between Clinical Characteristics and Quality of Life

Patients with severe CHD reported significantly lower scores in the domains of general health, role–physical, and physical functioning compared to patients with mild and moderate CHD. They also rated their overall health significantly worse on the EQ-VAS than patients with mild CHD and moderate CHD (*p* = 0.014 and *p* = 0.001, respectively) (Table 4). Similar results were observed when evaluating the influence of cyanosis on QoL. Cyanotic patients reported significantly lower scores compared to acyanotic patients in the same domains as patients with severe CHD and rated their overall health significantly worse on the EQ-VAS (*p* = 0.001).

Patients with heart failure (HF) reported significantly lower scores in most of the domains of the SF-36 questionnaire, including general health, role–physical, physical functioning, bodily pain, and social functioning compared to patients without HF. Their overall health assessment on the EQ-VAS was also significantly worse (*p* < 0.001).

## 4. Discussion

The QoL among ACHD patients in our cohort, as assessed by the SF-36 and EQ-5D questionnaires, was excellent in most domains. Several factors may contribute to these favourable outcomes. Most patients were diagnosed in early childhood and underwent appropriate interventions, followed by continuous, long-term medical surveillance aimed at early detection and management of potential complications. Furthermore, the majority of patients in our cohort had mild-to-moderate CHD, which is typically associated with fewer physical and functional limitations than severe CHD. Importantly, the chronic nature of the disease may also lead patients to adapt their lifestyle expectations over time, resulting in a perception of QoL that is comparable to that of their healthy peers [22,23].

The SF-36 questionnaire assesses both the physical and psychosocial aspects of QoL, including eight domains, the scores of which can be summarized into two composite scores, namely PCS and MCS [18]. We found overall higher scores in PCS compared to MCS, which differs from other similar studies that reported poor physical functioning and better results in the social and emotional domains [24,25]. This could be attributed to the fact that the majority of our patients had moderate or mild CHD, which generally have better exercise capacity. Nevertheless, general health and vitality were the worst-rated domains in our cohort, which is consisted with findings from other studies [10,26,27,28].

A substantial proportion of our patients—just under half—reported problems in the anxiety/depression domain of the EQ-5D, making it the most frequently affected area. These results are consistent with findings from the Slovenian population norms study for the EQ-5D, in which nearly 40% of individuals reported being at least slightly anxious or depressed [19]. Moreover, mood and anxiety disorders are also one of the most commonly reported comorbidities among ACHD patients [29,30,31,32,33,34]. This may be attributed to certain ACHD-specific factors, including burden of chronic disease, frequent interventions, and uncertainty about long-term prognosis [32,35,36,37]. The association between anxiety, depression, and reduced QoL is well established, highlighting the need for routine mental health screening and early, targeted interventions in this patient population [29].

HF is one of the most common complications in ACHD patients, with an estimated incidence of at least 30% in patients with complex CHD [38]. It is also the leading cause of premature death in this population [39,40]. Our findings are consistent with previous research showing that ACHD patients with HF experience significantly reduced QoL, not only in the domains of PCS, but also in the domain of social functioning [41,42]. Furthermore, results from the FRESH-ACHD registry indicate that lower QoL scores (e.g., SF-36 physical and general health domains) are associated with an increased risk of adverse outcomes within just one year, underlining the importance of integrating QoL assessments into routine clinical evaluation and rehabilitation strategies [42]. It is important to recognize that most ACHD patients have chronically abnormal baseline cardiovascular function due to the lifelong nature of their condition and have often adapted their daily activities to match their functional capacity [43]. As a result, QoL assessment in ACHD patients with HF may differ considerably from that in patients with acquired HF, as the former might require more frequent QoL evaluations to detect subtle but clinically meaningful changes over time. Additionally, the development of a QoL assessment tool specifically designed for ACHD-HF patients could offer more accurate and relevant insights into their unique physical and psychological challenges [44].

We acknowledge several limitations of our cross-sectional observational study. While the overall sample size was modest, particularly in the severe CHD subgroup, the findings provide valuable real-world insight into the QoL in this specific population as the study was conducted in a national reference centre for ACHD patients. However, we must recognize that our findings may not be fully generalizable to the broader ACHD population, due to selection bias. Specifically, as our study was conducted at a national reference centre, the cohort may not represent the full spectrum of ACHD patients, particularly those with mild conditions, who may not be referred to specialized centres. This could lead to an overrepresentation of patients with moderate and severe ACHD, who might experience different QoL outcomes compared to those in the general population with milder disease. Moreover, survivor bias is an additional limitation. Since our study only included patients who had survived to adulthood, data from individuals with more severe forms of CHD who may not have survived to this age are not contained in the study, which could lead to an overestimation of QoL outcomes. As highlighted in previous studies on QoL in this population, a key methodological challenge remains the absence of a standardized definition and measurement tool for QoL. This lack of uniformity poses significant difficulties for the interpretation and comparison of QoL findings across studies and underscores the need for consensus in future research. Moreover, QoL instruments used in this study are generic, which may not fully reflect disease-specific challenges faced by ACHD patients, such as concerns related to reproduction, employment, and uncertainty about the future. Future research would benefit from the inclusion of ACHD-specific tools, such as the ACHD-QoL questionnaire, to capture these important aspects more accurately.

## 5. Conclusions

QoL in our cohort of ACHD patients, assessed with the SF-36 and EQ-5D questionnaires, was generally excellent across most domains. However, MCS scores were significantly lower than PCS scores, and nearly half of the patients reported difficulties in the anxiety/depression domain. These findings underscore the importance of prioritizing mental health and psychological well-being as key components of care in this population. Furthermore, ACHD patients with established HF reported lower QoL scores across most domains, highlighting the need for closer clinical monitoring and the development of dedicated QoL assessment tools tailored to the specific needs of this complex and heterogeneous group.

## Figures and Tables

**Table 1:** Baseline characteristics.

	All Patients (*n* = 123)
Male sex, *n* (%)	54 (43.9%)
Age, median (Q1–Q3)	34 (29–41) years
Marital status, *n* (%)	
o Single	38 (30.9%)
o Married or long-term relationship	82 (66.7%)
o Divorced or widowed	3 (2.4%)
Education level, *n* (%)	
o Less than high school	11 (8.9%)
o High school	48 (39.0%)
o Vocational school or university degree	64 (52.0%)
Employment status, *n* (%)	
o Part-time or full-time work	99 (80.5%)
o Student	5 (4.1%)
o Retired	9 (7.3%)
o Unemployed	10 (8.1%)

**Table 2:** Clinical data.

	All Patients (*n* = 123)
BMI (kg/m^2^)	24.0 ± 4.0
NYHA class	
o I	72 (59%)
o II	34 (28%)
o III	17 (14%)
o IV	0
Past medical history, *n* (%)	
o Arterial hypertension	33 (26.8%)
o Arrhythmias	60 (48.8%)
o CIED	10 (8.1%)
CHD complexity, *n* (%)	
o Mild	29 (23.6%)
o Moderate	75 (61.0%)
o Severe	19 (15.4%)
Cyanosis, *n* (%)	18 (14.6%)

Abbreviations: BMI, body mass index; NYHA, New York Heart Association; CIED, cardiac implantable electronic devices; CHD, congenital heart disease.

**Table 3:** Quality of life results according to the SF-36 questionnaire and the EQ-5D questionnaire.

		SF-36 Domain, Median (Q1–Q3)					All Patients (*n* = 123)				
		Physical functioning					90.0 (75.0–100)				
		Role–physical					100 (75.0–100)				
		Bodily pain					90.0 (77.5–100)				
		General health					70.0 (50.0–80.0)				
		Vitality					70.0 (55.0–80.0)				
		Social functioning					100 (75.0–100)				
		Role–emotional					100 (66.0–100)				
		Mental health					80 (72.0–88.0)				
Scales		0–10	11–20	21–30	31–40	41–50	51–60	61–70	71–80	81–90	91–100
EQ-VAS	N %	0 0	0 0	1 0.8	1 0.8	3 2.4	5 4.1	26 21.1	27 22.0	43 35.0	17 13.8
Domains of EQ-5D											
Problems		Extreme		Severe		Moderate		Slight		No	
Mobility	N %	0 0		0 0		4 3.2		16 13.0		103 83.7	
Self-care	N %	0 0		0 0		0 0		8 6.5		115 93.5	
Usual activities	N %	0 0		0 0		5 4.1		22 17.9		96 78.0	
Pain/discomfort	N %	0 0		0 0		3 2.4		17 13.8		103 83.7	
Anxiety/depression	N %	0 0		2 1.6		9 7.3		37 30.0		75 61.0	

Abbreviations: SF-36, Short Form–36.

**Table 4:** Correlations between clinical characteristics and quality of life.

	Severe CHD	Cyanosis	Heart Failure
SF-36 domain			
Physical functioning	***p*** < 0.001 * ***p*** ** < 0.001 ^†^**	***p*** ** < 0.001 ^‡^**	***p*** ** < 0.001 ^a^**
Role–physical	***p*** = 0.001 * ***p*** ** = 0.003 ^†^**	***p*** ** = 0.002 ^‡^**	***p*** ** < 0.001 ^a^**
Bodily pain	*p* = 0.163	*p* = 0.219 ^‡^	***p*** ** = 0.028 ^a^**
General health	***p*** = 0.012 * ***p*** ** = 0.001 ^†^**	***p*** ** = 0.001 ^‡^**	***p*** ** = 0.011 ^a^**
Vitality	*p* = 0.891	*p* = 0.886 ^‡^	*p* = 0.648 ^a^
Social functioning	*p* = 0.324	*p* = 0.498 ^‡^	***p*** ** = 0.038 ^a^**
Role–emotional	*p* = 0.221	*p* = 0.514 ^‡^	*p* = 0.310 ^a^
Mental health	*p* = 0.744	*p* = 0.911 ^‡^	*p* = 0.684 ^a^
EQ-VAS	***p*** = 0.014 * ***p*** ** = 0.001 ^†^**	***p*** ** = 0.001 ^‡^**	***p*** ** < 0.001 ^a^**

Abbreviations: CHD, congenital heart disease; SF-36, Short Form–36; EQ-VAS, Euro Quality of Life–Visual Analogue Scale. *p* values < 0.05 are indicated in bold. * indicates the difference between patients with mild CHD and severe CHD, and ^†^ indicates the difference between moderate CHD and severe CHD. ^‡^ indicates the difference between cyanotic and acyanotic patients. ^a^ indicates the difference between patients with heart failure and patients without heart failure.

## Data Availability

The data presented in this study are available upon reasonable request from the corresponding author.
